# Relationship of life-meaning orientations, depression, anxiety and stress among patients living with HIV during the second wave of the COVID-19 pandemic

**DOI:** 10.1192/j.eurpsy.2023.1722

**Published:** 2023-07-19

**Authors:** V. I. Rozhdestvenskiy, V. V. Titova, I. A. Gorkovaya, D. O. Ivanov, Y. S. Aleksandrovich

**Affiliations:** Department of Psychosomatics and Psychotherapy, Saint Petersburg State Pediatric Medical University, Saint Petersburg, Russian Federation

## Abstract

**Introduction:**

The pandemic is an undeniably stressful factor on a planetary scale. Life meaning, specific meaning-life orientations, and aspects of locus of control mediate one’s relationship to one’s life circumstances. Thus, the noetic part of human existence can relate to the perception of the pandemic.

**Objectives:**

The study aimed to examine the relationship between life-meaning orientations and nonspecific emotional reactions in HIV-infected patients during the second wave of the pandemic.

**Methods:**

The data were collected from February to July 2021 using a Google form we developed. Fifty-nine HIV-positive patients participated in the study. We used the Purpose-in-Life Test to examine life-meaning orientations and the DASS-21 to diagnose depression, anxiety, and stress. Both questionnaires were adapted for use in Russia.

**Results:**

We obtained the following mean values on the PiLT scales: “general index of life meaningfulness” — M = 94.39±19.71; “goals in life” — M = 30.80±7.75; “life process” — M = 26.93±6.66; “life performance” — M = 23.69±6.66; “locus of control — Me” — M = 19. 61±5.05; “locus of control — life” — M = 25.90±7.43. All PiLT scales had statistically significant negative correlations with depression, anxiety, and stress, except “life process,” which was not associated with anxiety (r_xy_ = -0.215, p > 0.05).

**Conclusions:**

In the COVID-19 pandemic, life meaningfulness, ability to manage life, and freedom of choice may be considered as predictors of emotional well-being among patients living with HIV. The method of the existential psychotherapy can be effective apply among this group of patients.

**Disclosure of Interest:**

None Declared

